# Supplementary material for: Microbial reduction of metal-organic frameworks enables synergistic chromium removal
Source: Nat Commun. 2019 Nov 18;10:5212. doi: 10.1038/s41467-019-13219-w (PMC6861306; doi:10.1038/s41467-019-13219-w)
Supplement: Supplementary file 1 — Supplementary Information [file 41467_2019_13219_MOESM1_ESM.pdf]

Supplementary Information

Microbial Reduction of Metal-Organic Frameworks Enables Synergistic Chromium Removal

Springthorpe et al.

Supplementary Methods

Chemicals and reagents

Trimesic acid (H_3BTC, Sigma-Aldrich, 95%), sodium hydroxide (NaOH, VWR, pellets), ferrous chloride tetrahydrate ($FeCl_2 \cdot 4H_2O$, JT Baker), ferric chloride hexahydrate ($FeCl_3 \cdot 6H_2O$, Fisher-Scientific, ACS grade), fumaric acid ($HO_2CCHCHCO_2H$, Alfa Aesar, 99%), red hematite (Fe_2O_3, Strem Chemicals, 99.8% Fe), magnesium oxide (MgO, Alfa Aesar), oxalic acid ($C_2H_2O_{4-}$, VWR, 10% w/v), HEPES buffer (4-(2-hydroxyethyl)-1-piperazineethanesulfonic acid, IBI Scientific), potassium phosphate dibasic (K_2HPO_4, Sigma-Aldrich), potassium phosphate monobasic (KH_2PO_4, VWR), sodium chloride (NaCl, VWR), ammonium sulfate $(NH_4)_2SO_4$, Fisher Scientific), magnesium(II) sulfate heptahydrate ($MgSO_4 \cdot 7H_2O$, Sigma-Aldrich), EDTA acid disodium salt dihydrate ($C_{10}H_{14}N_2Na_2O_8 \cdot 2H_2O$, VWR), manganese(II) sulfate monohydrate ($MnSO_4 \cdot H_2O$, VWR), ferrous sulfate heptahydrate ($FeSO_4 \cdot 7H_2O$, Alfa Aesar), cobalt(II) nitrate hexahydrate ($Co(NO_3)_2 \cdot 6H_2O$, Strem Chemicals), calcium chloride dihydrate ($CaCl_2 \cdot 2H_2O$, Sigma-Aldrich), zinc(II) sulfate monohydrate ($ZnSO_4 \cdot H_2O$, Strem Chemicals), cupric sulfate pentahydrate ($CuSO_4 \cdot 5H_2O$, VWR), aluminum potassium sulfate ($AlK(SO_4)_2$, Acros Organics), boric acid (H_3BO_3, VWR), sodium molybdate dihydrate ($Na_2MoO_4 \cdot 2H_2O$, Beantown Chemical), sodium selenite (Na_2SeO_3, Acros Organics), sodium tungstate dihydrate ($Na_2WO_4 \cdot 2H_2O$, Alfa Aesar), nickel(II) chloride hexahydrate ($NiCl_{-2} \cdot 6H_2O$, Alfa Aesar), Syto™ 9 green fluorescent nucleic acid stain (ThermoFisher Scientific), SYPRO™ Ruby protein gel stain (ThermoFisher Scientific), Better Bradford Reagent (ThermoFisher Scientific) and casamino acids (VWR) were used as received. Sodium DL-lactate ($C_3H_5NaO_3$, VWR, 60% in water) was filtered using 0.2 μm PES filters. Sodium fumarate ($Na_2C_4H_2O_4$, VWR) was diluted in H_2O and then filtered using 0.2 μm PES filters. LB Lennox agar powder (VWR) was dissolved in H_2O and sterilized at 121°C and 100 kPa for 1 h. Hydrochloric acid (HCl, Sigma-Aldrich, 37%) was diluted in H_2O prior to use. Ferrozine (3-(2-pyridyl)-5,6-bis(4-sulfophenyl)-1,2,4-triazine disodium salt hydrate, TCI Chemicals), ammonium acetate ($NH_4CH_3CO_2$, VWR), hydroxylamine hydrochloride ($HONH_2 \cdot HCl$, Alfa Aesar, ACS grade), potassium dichromate ($K_2Cr_2O_7$, Alfa Aesar, ACS grade), 1,5-diphenylcarbazide (DPC, Alfa Aesar), and acetone ($(CH_3)_2CO$, Fisher Scientific, ACS grade) were used as received. Sulfuric acid (H_2SO_4, VWR, 95-97%) was diluted in H_2O before use. Iron(III) citrate ($FeC_6H_5O_7$, Alfa Aesar, Fe(III) 16.5-20%, Fe(II) max 5%) was used as received. Deuterium oxide (D_2O, Sigma-Aldrich, 99.9%) was used for NMR as received. Nitric acid (HNO_3, Sigma-Aldrich, trace mineral grade) was diluted to 2% for ICP-MS analysis. Ultrapure water was produced by a MilliQ Integral Water Purification System.

Powder X-Ray Diffraction

PXRD was performed using a Rigaku R-Axis Spider X-Ray Diffractometer with curved image plate detector. The as-synthesized metal-organic frameworks were analyzed without modification. Following reduction of the materials by MR-1, the abiotic and biotic samples were washed with fresh H_2O to remove any residual salts from the medium and stored in anaerobic conditions prior to analysis. Immediately before analysis, the samples were coated in mineral oil to prevent oxidation in the aerobic environment of the instrument. A sample of mineral oil run under the same instrument conditions was subtracted as background from the spectra.

Surface Area Measurements

Langmuir surface area measurements were conducted using N_2 (99.999%) gas adsorption collected on a Micromeritics ASAP 2020 Physisorption instrument. Approximately 60 mg of either Fe-BTC, MIL-100 or MIL-88A were activated under high vacuum at 120 °C with a ramp of 0.1 deg/min for 24 h before analysis.

Electron Microscopy

SEM was used to determine the extent of aggregation in the as-synthesized metal-organic framework. Images were collected using Zeiss Supra 40V SEM. TEM was used to determine particle morphology of cycled MIL-100. The TEM grids used for STEM and element mapping were imaged using a Thermo Fisher Tecnai TEM.

Leaching and Framework Stability Analysis

The metal-organic frameworks were tested for leaching and framework stability by exposing the materials to culture conditions for 48 h. The ferrozine assay was used to determine the Fe(III) concentration in the supernatants after the metal-organic frameworks ([Fe(III)=15mM). For Fe(II) analysis, samples were acidified with 6M HCl in a 1:1 ratio prior to analysis with the ferrozine assay. For total Fe analysis, the sample was mixed with 1 M hydroxylamine hydrochloride in a 1:1 ratio and analyzed by the ferrozine assay. Fe(III) concentrations were calculated by subtracting the Fe(II) concentration from the total Fe concentration.

NMR spectroscopy was used to determine the extent of fumarate leaching from MIL-88A ([$Fumarate^-$]=5 mM) in 1 mL of D_2O and stored anaerobically at 30 °C. After 48 h, the samples were centrifuged and the supernatant was removed. The supernatant was spiked with 1.0 mg of benzoic acid as an internal standard. A standard of fumaric acid (1.0 mg/mL) and benzoic acid (1.0 mg/mL) was mixed in 1 mL D_2O. An aliquot

of the supernatant (50 μL) was diluted in 950 μL of D_2O and analyzed on an Agilent MR400 NMR (400 MHz).

Framework structural stability was tested by exposing the metal-organic frameworks to culture conditions for 48 h. Following exposure, the materials were washed and analyzed by PXRD.

Nucleic Acid and Extracellular Protein Staining

To assess biomass accumulation of *S. oneidensis* MR-1, nucleic acids and extracellular proteins were stained with fluorescent dyes. Cultures were prepared in SBM with 20 mM lactate and either MIL-100 or ferrihydrite ([Fe(III)]=15 mM). To improve the fluorescent signal, the inoculating concentration of washed cells was increased OD_{600}=0.02. Cultures were anaerobically incubated at 30 °C for 48 hours. For the staining of bacterial nucleic acids, 200 μL of the culture suspension was removed and pelleted before removing the supernatant. The pellet was resuspended in 100 μL of 0.85% NaCl solution, mixed with 2X Syto™ 9 solution in a 96 well plate, and incubated at room temperature for 15 minutes in the dark. Fluorescence was measured with an excitation/emission of 485/530 nm using a BMG LabTech CLARIOstar Monochromator Microplate Reader. For the staining of extracellular proteins, 100 μL of the culture suspension was removed and pelleted prior to removing the supernatant. The pellet was mixed with 200 μL of 1X SYPRO™ Ruby fluorescent dye in a 96 well plate. The plate was incubated in the dark at room temperature for 15 minutes before measure fluorescence at an excitation/emission of 460/640 nm using a plate reader. The nucleic acid fluorescence and extracellular fluorescence for biotic samples was corrected by subtracting the abiotic measurement.

Reduction of MIL-100 by *E. coli*

To demonstrate that the reduction of MIL-100 is not due to promiscuous reduction, a culture of MIL-100 ([Fe(III)]=15 mM) and 20 mM lactate in SBM was inoculated with stationary-phase *E. coli* (OD_{600}=0.002). *E. coli* MG1655, generously provided by Dr. Lydia Contreras (University of Texas, Austin, TX), was pregrown anaerobically in 40 mM fumarate and 20 mM lactate in SBM, washed twice and diluted to an OD_{600}=0.2 prior to culture inoculation. All cultures were incubated at 37 °C in an anaerobic environment. Fe(II) concentrations were analyzed using the ferrozine assay. An abiotic control was also tested. Experiments were performed in triplicate.

ICP-MS analysis

ICP-MS was used to analyze total Cr in the supernatant of biotically-reduced MIL-100 challenged with 0.5 mM Cr(VI). Cultures containing MIL-100 ([Fe(III)=15 mM) and 20 mM lactate were inoculated with MR-

1 (OD_{600}=0.002, anaerobic pregrowth). MR-1 reduced MIL-100 until the [Fe(II)] = 2.8 mM after which cultures were challenged with 0.5 mM Cr(VI) and aliquots of the supernatant were removed for analysis. Samples were centrifuged (10000 x g, 1 min), frozen in liquid N_2 and stored at -20°C until further sample preparation. For the ICP-MS sample preparation, the samples were thawed and diluted 200-fold in 2% HNO_3 immediately prior to analysis. A 7500ce Agilent ICP-MS was used to analyze total Cr (LOD:0.54 ppb) and was verified using m/Z=52 and 53.

MR-1 reduction of Cr(VI)

Reduction of 100 µM Cr(VI) by MR-1 was tested by inoculating a culture of 20 mM lactate and 50 µM $K_2Cr_2O_7$ in SBM with stationary phase MR-1 (OD_{600}=0.002, anaerobic pregrowth). Cultures were stored at 30 °C in anaerobic conditions for 24 h. Cr(VI) concentrations were analyzed using the DPC assay. An abiotic control was also tested.

Cr(VI) reduction by $FeCl_2$

Cr(VI) reduction by abiotic Fe(II) was tested by mixing a solution of $FeCl_2 \cdot 4 \cdot H_2O$ (either 0.5 mM, 1.0 mM or 2.0 mM), 20 mM lactate and 0.5 mM Cr(VI) in SBM (pH=7.2). Solutions were stored in anaerobic conditions at 30 °C for 24 h. Cr(VI) concentrations were determined using the DPC assay.

Cr(VI) reduction by MR-1 reduced Fe(III)-citrate

Cr(VI) reduction by biotically reduced Fe(II) was tested by inoculating a culture of Fe(III)-citrate (5 mM) and 20 mM lactate in SBM with stationary phase MR-1 (OD_{600}=0.002, anaerobic pregrowth). MR-1 reduced Fe(III)-citrate until Fe(II) concentrations for 8 h, or when Fe(II) concentrations were comparable to those observed at 24 h with MR-1 and MIL-100. Fe(II) concentration in the Fe(III)-citrate and MR-1 cultures was 2.5 mM after 8 h. Once Fe(III) reduction was established, the cultures were challenged with 0.5 mM Cr(VI). After 12 h, the cultures were challenged again with 0.5 mM Cr(VI). Fe(II) and Cr(VI) concentrations were monitored throughout the experiment using the ferrozine and DPC assays, respectively. An abiotic control was also tested using the same procedure.

Supplementary Figures

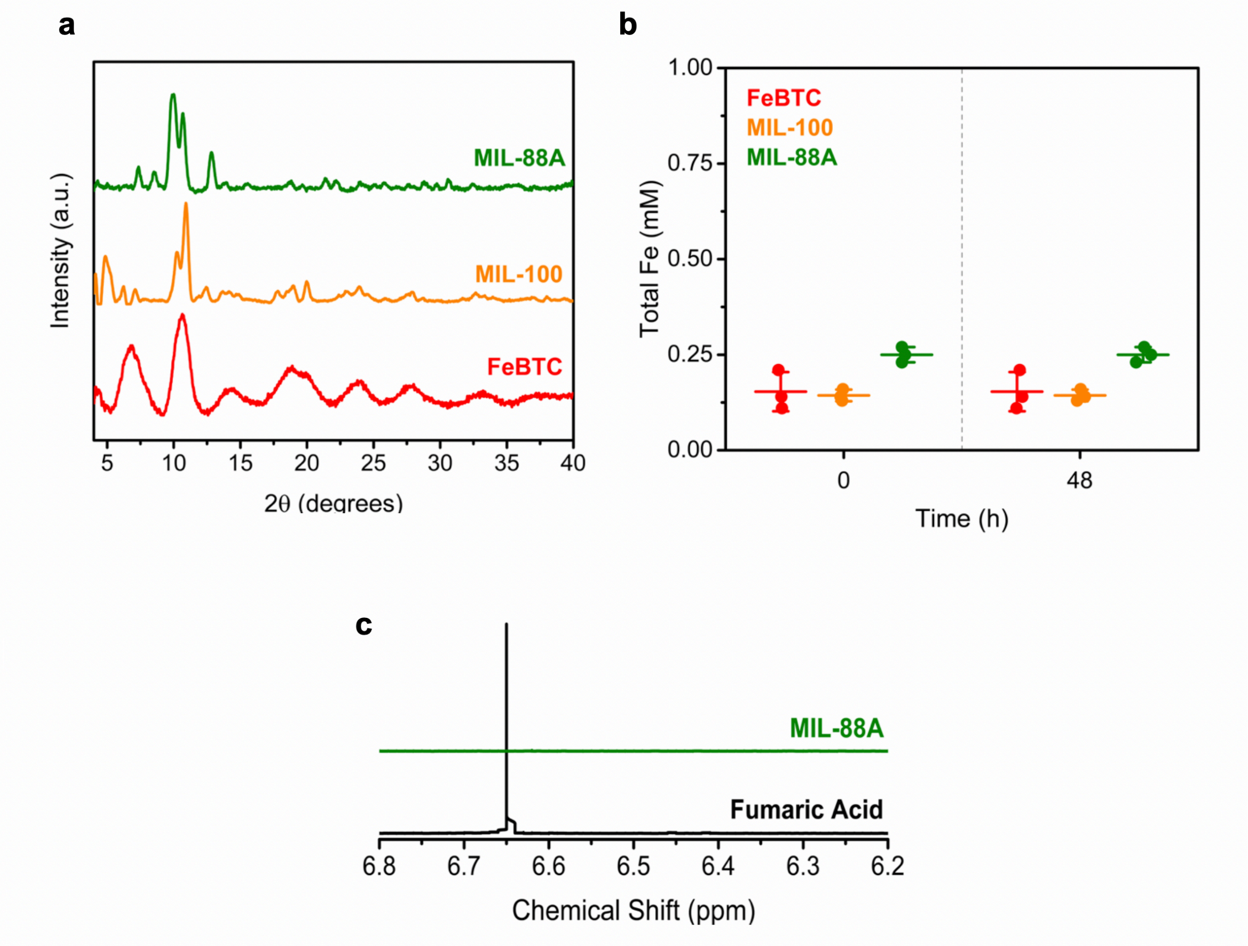

Supplementary Figure 1. Stability of metal-organic frameworks. (a) PXRD spectra of Fe-BTC, MIL-100, and MIL-88A after exposure to standard culture conditions for 48 h (SBM, 20 mM lactate 30 ºC). (b) [Fe(III)] in the supernatant of Fe-BTC, MIL-100, and MIL-88A after 48 h. Data show mean ± S.D. for three independent replicates. (c) ^1H NMR of the MIL-88A supernatant after 48 h and a 1 mg/mL fumaric acid standard (limit of detection: 18.6 μM). Source data for (a)-(c) are provided as a Source Data file.

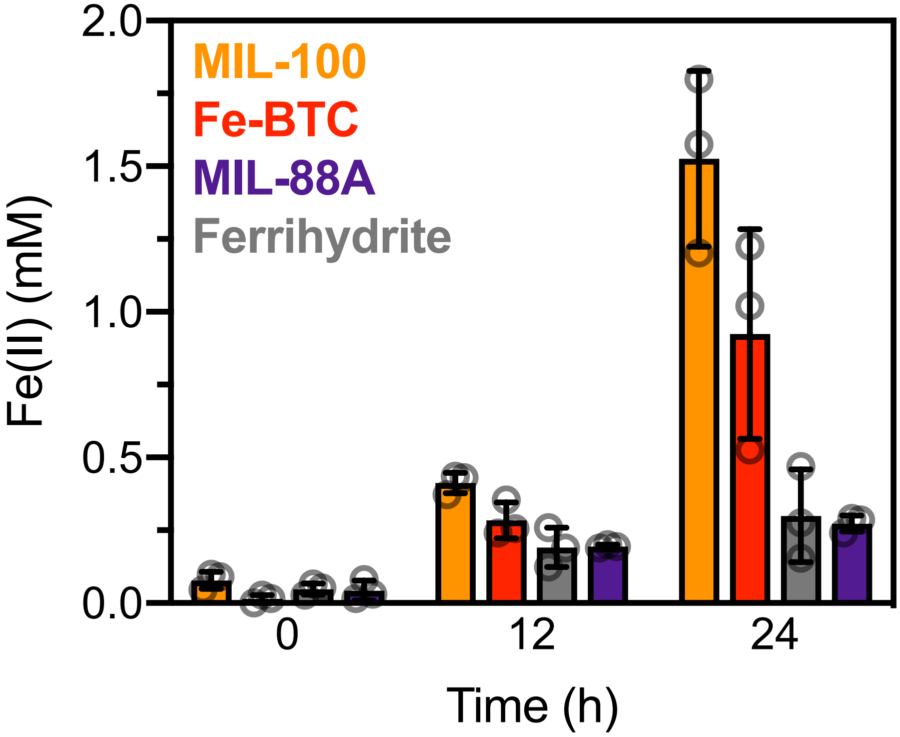

Supplementary Figure 2. Concomitant Fe(III) reduction with CFU counts. Fe(II) concentrations of cultures of *S. oneidensis* MR-1 (inoculating OD_{600}=0.002) in SBM with 20 mM lactate and either MIL-100, Fe-BTC, MIL-88A, or ferrihydrite. Data show mean ± S.D. for three independent biological replicates. These data were collected concomitantly with growth (as measured by CFU) shown in Figure 2a. Source data are provided as a Source Data file.

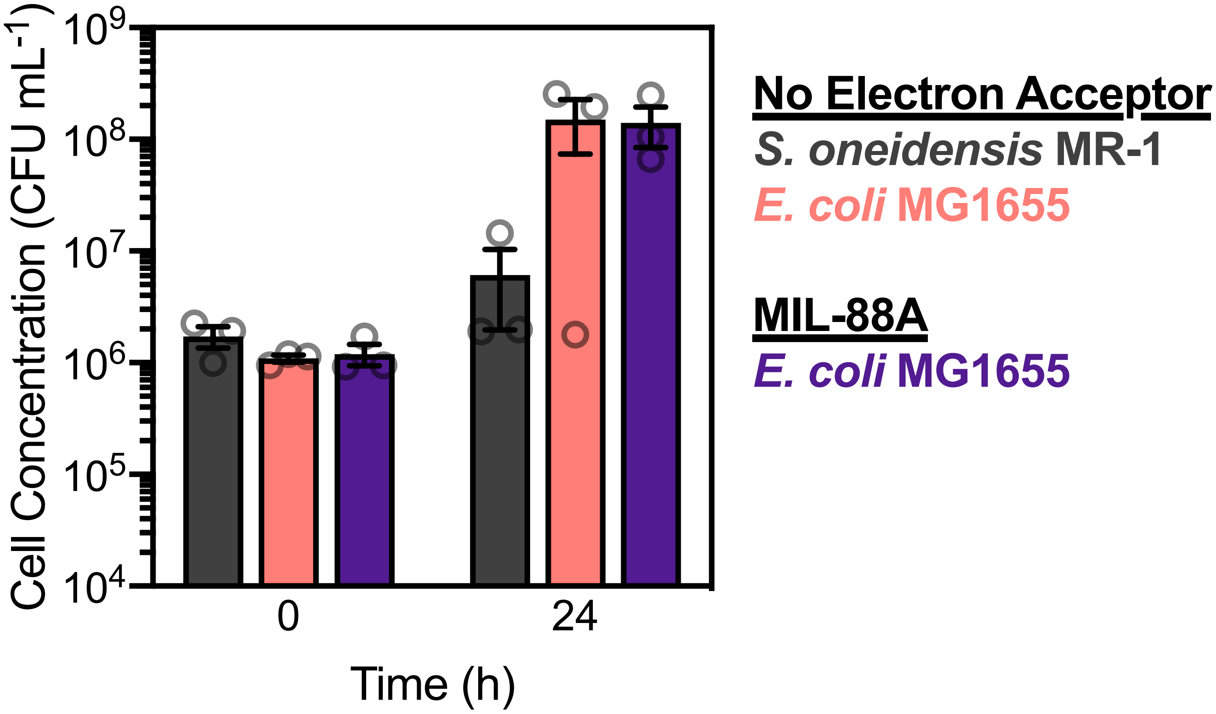

Supplementary Figure 3. Growth on MIL-88A. CFU counts of *S. oneidensis* MR-1 and *E. coli* MG1655 with no electron acceptor present (only SBM and lactate), as well as MG1655 with MIL-88A as an electron acceptor. The initial inoculating OD_{600}=0.002. Data show mean ± S.E.M. for three independent biological replicates. Source data are provided as a Source Data file.

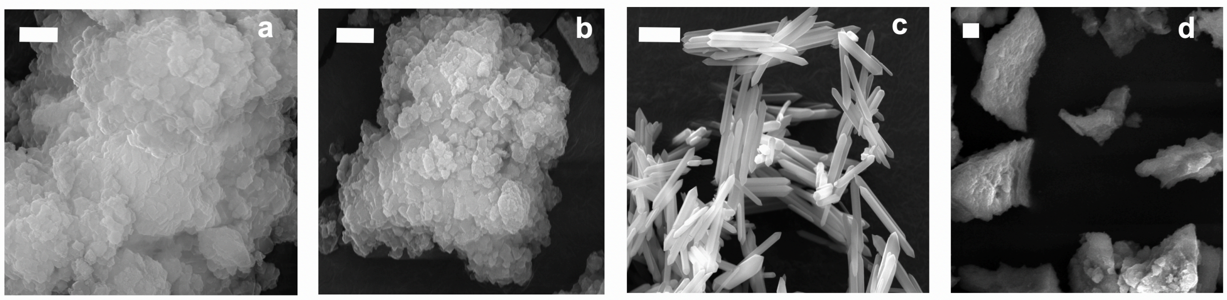

Supplementary Figure 4. Morphology of metal-organic frameworks and ferrihydrite. SEM images of (a) MIL-100, (b) Fe-BTC, (c) MIL-88A, and (d) ferrihydrite, as synthesized. Scale bars are 2 µm.

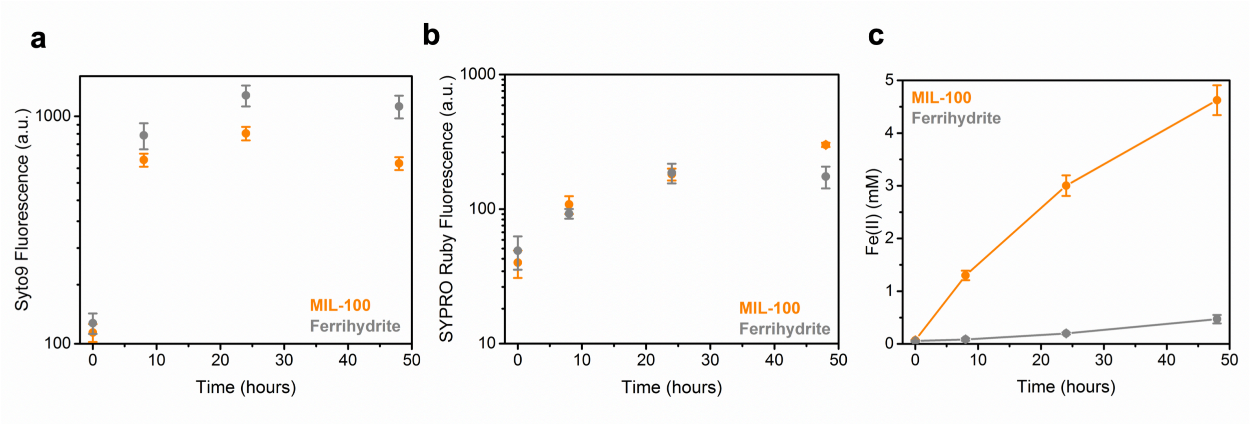

Supplementary Figure 5. Nucleic acid and biofilm matrix protein quantification. (a) Measure of nucleic acids (Syto™ 9) for *S. oneidensis* MR-1 (inoculating OD_{600}=0.02) grown on MIL-100 and ferrihydrite over 48 h. Data show mean ± S.E.M. for four independent biological replicates. (b) Measure of biofilm matrix proteins (SYPRO™ Ruby) for *S. oneidensis* MR-1 (inoculated OD_{600}=0.02) grown on MIL-100 and ferrihydrite over 48 h. Data show mean ± S.D. for four independent biological replicates. (c) Fe(III) reduction by MR-1 grown on MIL-100 and ferrihydrite over 48 h, measured in parallel with Syto™ 9 and SYPRO™ Ruby. The initial inoculating OD_{600}=0.02. Data show mean ± S.D. for four independent biological replicates. Source data for (a)-(c) are provided as a Source Data file.

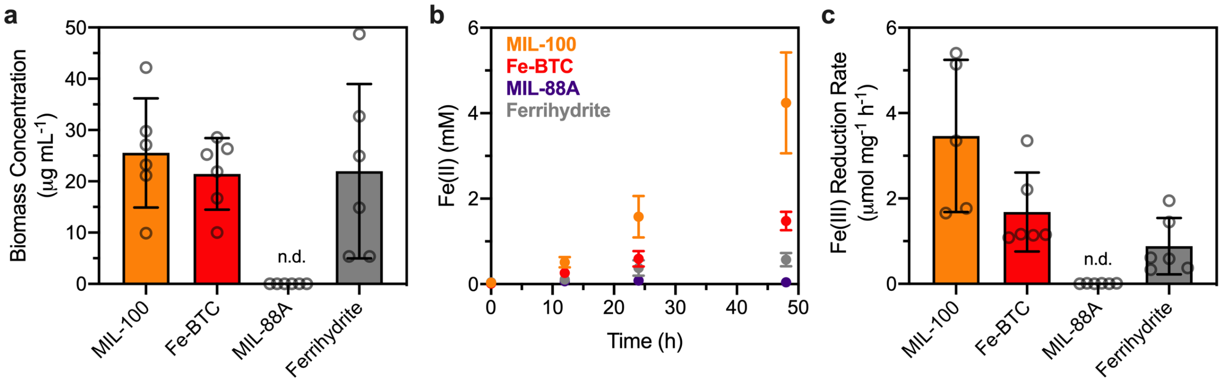

Supplementary Figure 6. Normalized Fe(III) reduction rates after 48 h. (a) Total protein concentrations of *S. oneidensis* MR-1 (inoculating OD_{600}=0.002) grown on MIL-100, Fe-BTC, MIL-88A, and ferrihydrite after 48 h. Data shown are mean ± S.D. for six independent replicates. (b) Total Fe(II) concentrations in suspensions containing *S. oneidensis* MR-1 (inoculating OD_{600}=0.002) and either MIL-100, Fe-BTC, MIL-88A, or ferrihydrite over 48 h. Data shown are mean ± S.D. for six independent replicates. (c) Total protein normalized Fe(III) reduction rates for *S. oneidensis* MR-1 grown on MIL-100, Fe-BTC, MIL-88A, and ferrihydrite at 48 h. n.d. indicates the rate could not be determined due to lack of detectable biomass. Data shown are mean ± S.D. for at least 5 independent replicates. Source data for (a)-(c) are provided as a Source Data file.

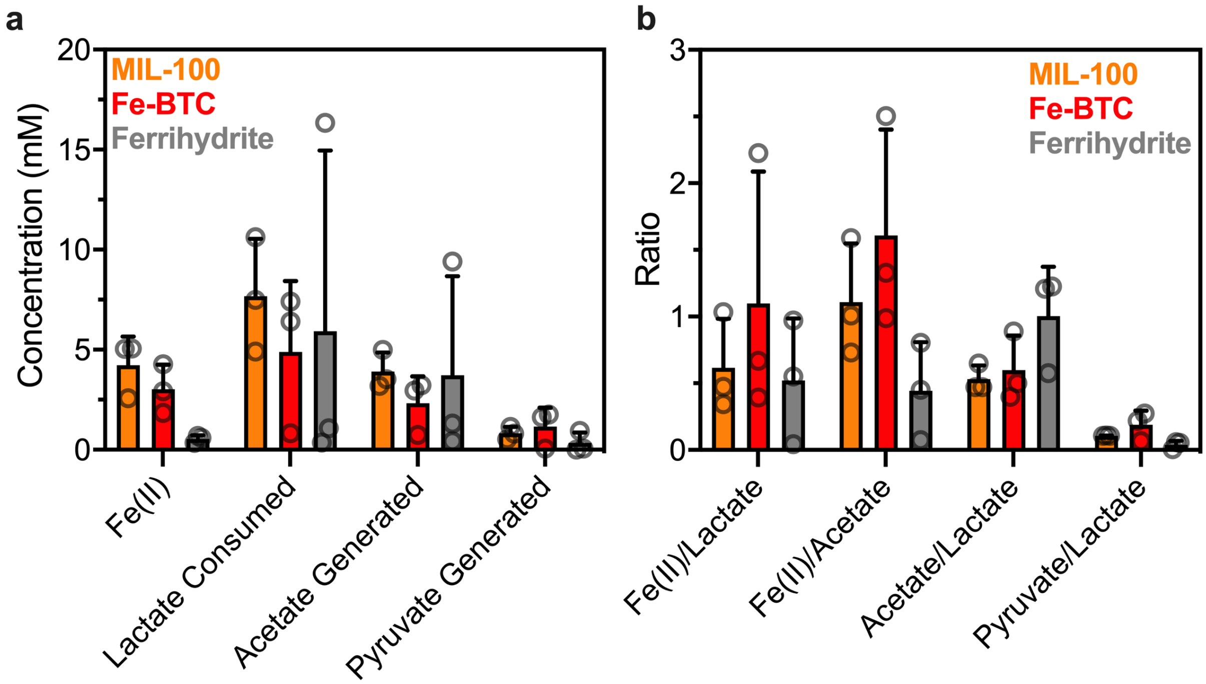

Supplementary Figure 7. Metabolite quantification. (a) Concentrations of Fe(II), lactate consumed, acetate generated and pyruvate generated in *S. oneidensis* MR-1 cultures after 48 hours (inoculating OD_{600}=0.002) and MIL-100, Fe-BTC, or ferrihydrite. Data shown are mean + S.D. for three independent replicates. (b) The ratios of utilized and generated metabolites within replicates. Data shown are mean + S.D. for three independent replicates. Source data for (a) and (b) are provided as a Source Data file.

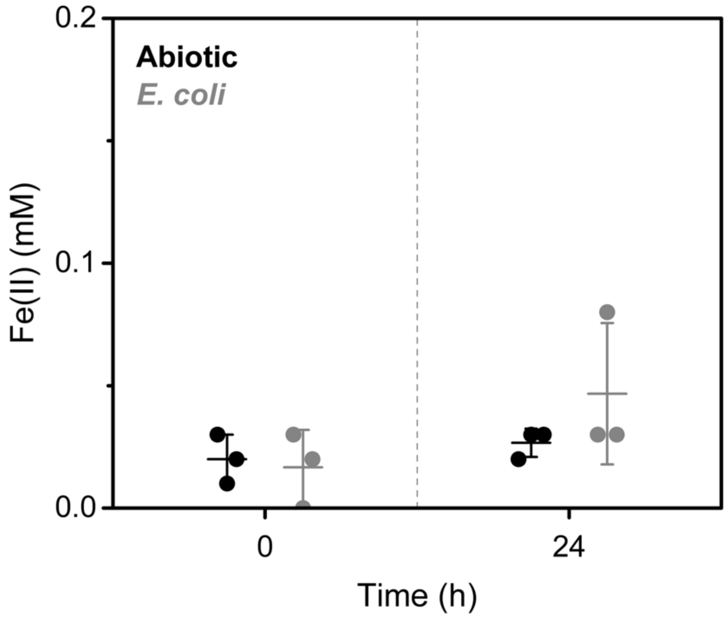

Supplementary Figure 8. *E. coli* reduction of MIL-100. Reduction of Fe(III) by *E. coli* (inoculating OD_{600}=0.002) when cultured with MIL-100 ($[Fe(III)]_0$=15 mM) and 20 mM lactate in SBM. Data show mean ± S.D. for three independent replicates. Source data are provided as a Source Data file.

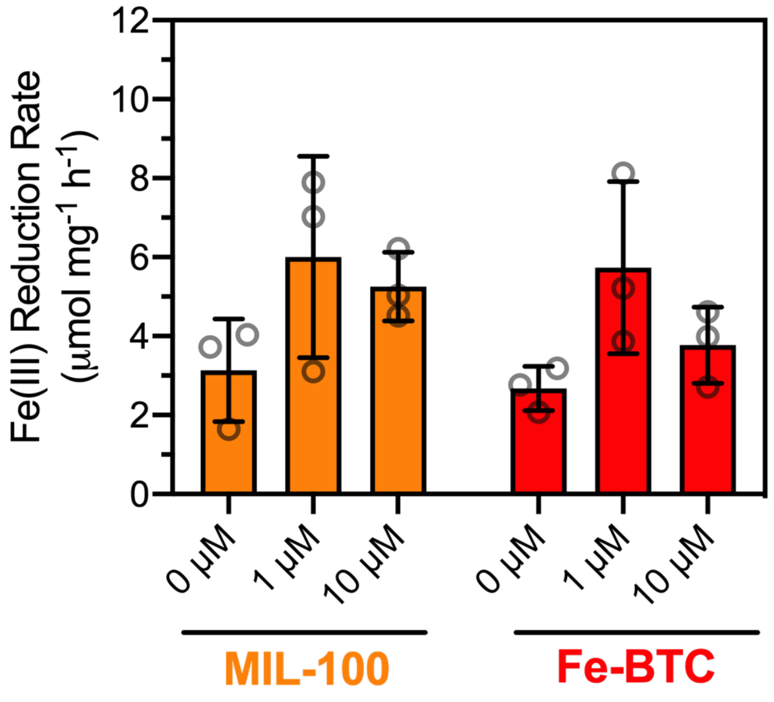

Supplementary Figure 9. Normalized Fe(III) reduction rate with exogenous riboflavin. Total protein normalized Fe(III) reduction of MIL-100 and Fe-BTC by *S. oneidensis* MR-1 with 0 µM, 1 µM, and 10 µM exogenous riboflavin. Data show mean ± S.D. for three independent experiments. Source data are provided as a Source Data file.

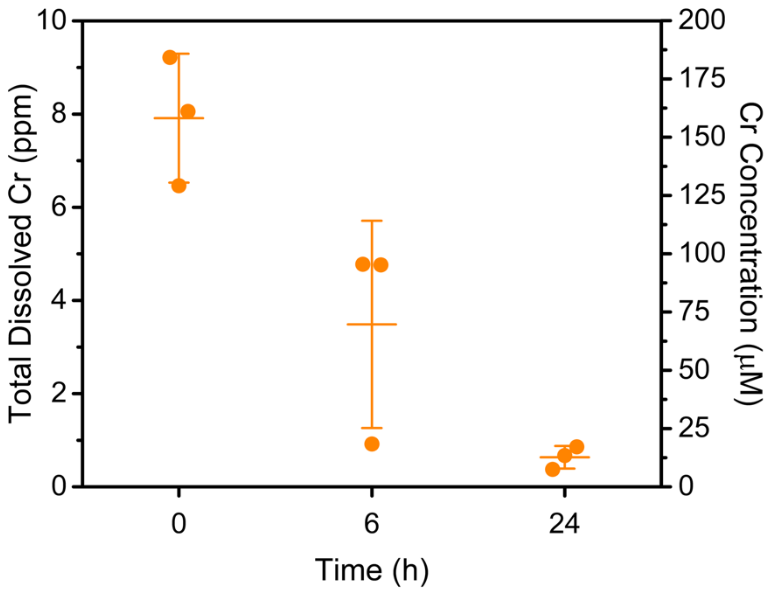

Supplementary Figure 10. Total Cr removal. Total Cr, as measured by ICP-MS, in the supernatant of MR-1 reduced MIL-100 after a challenge with 0.5 mM Cr(VI). Data show mean ± S.D. for three independent biological replicates. Source data are provided as a Source Data file.

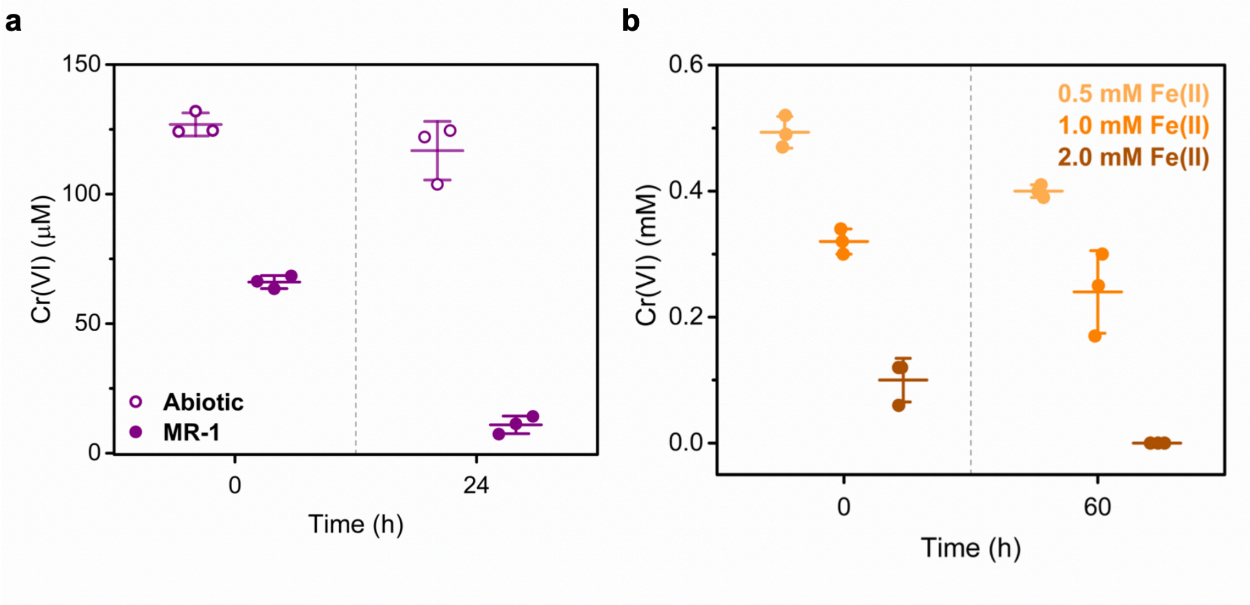

Supplementary Figure 11. Cr(VI) reduction by *S. oneidensis* and Fe(II). (a) Reduction of 100 μM Cr(VI) by *S. oneidensis* MR-1 and (b) reduction of 0.5 mM Cr(VI) by $FeCl_2 \cdot 4H_2O$ (either 0.5 mM, 1.0 mM, or 2.0 mM). Data show mean ± S.D. for three independent replicates. Source data for (a) and (b) are provided as a Source Data file.

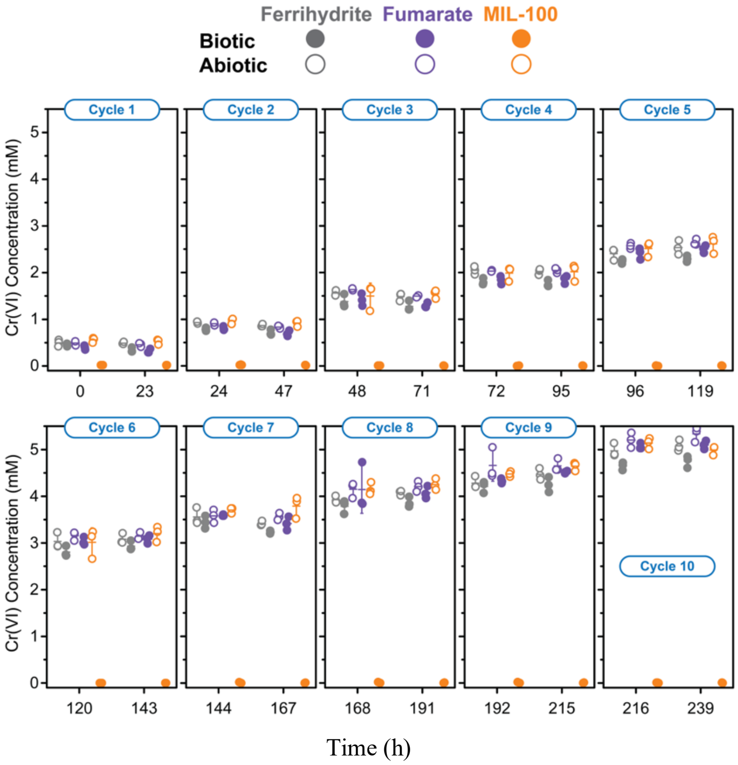

Supplementary Figure 12. Cr(VI) cycling. Cr(VI) concentrations in the supernatant for each Cr(VI) challenge (1-10) for MIL-100 (*orange*), ferrihydrite (*grey*), and fumarate (*purple*). Data show mean ± S.D. for three independent replicates. Source data are provided as a Source Data file.

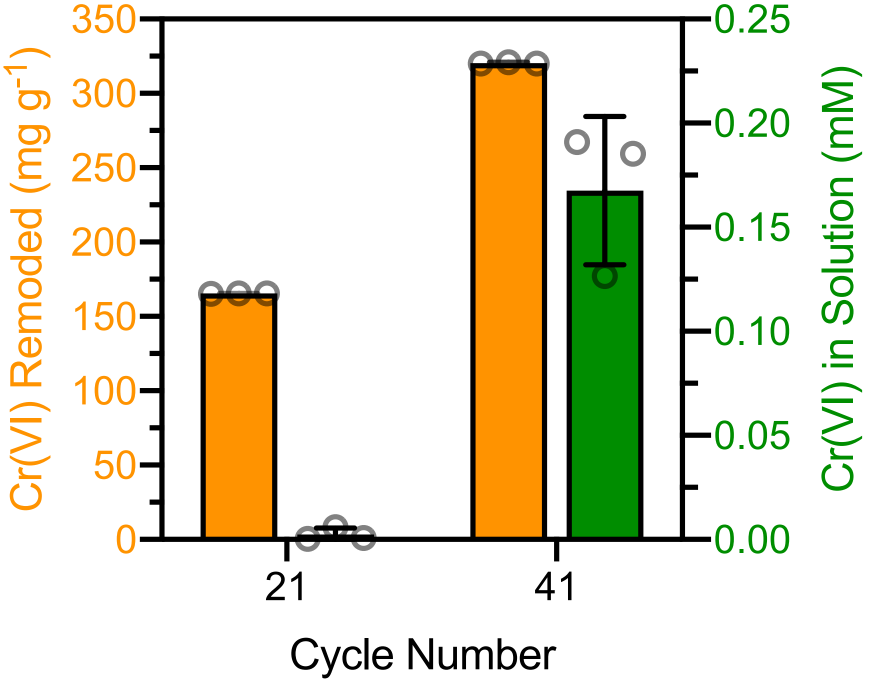

Supplementary Figure 13. Cr(VI) removal for long-term cycling. Cr(VI) remaining in the supernatant and total Cr(VI) removed for biotic MIL-100 samples after 21 and 41 additions of 0.5 mM Cr(VI). Data show mean ± S.D. for three independent replicates. Source data are provided as a Source Data file.

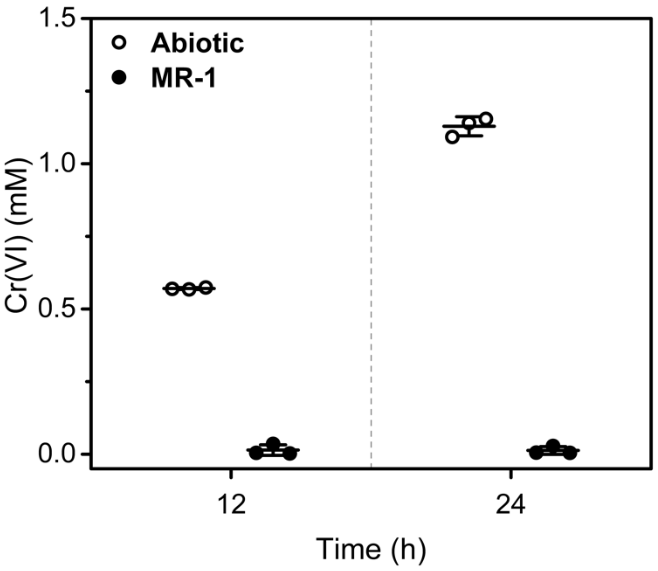

Supplementary Figure 14. Fe(II) reduction of Cr(VI). Reduction of 0.5 mM Cr(VI) by *S. oneidensis* MR-1 reduced Fe(III)-citrate (2 cycles). *S. oneidensis* MR-1 was grown on Fe(III)-citrate for ca. 12 h prior to initial Cr(VI) addition. A second dose of Cr(VI) was added at 24 h. Data show mean ± S.D. for three independent replicates. Source data are provided as a Source Data file.

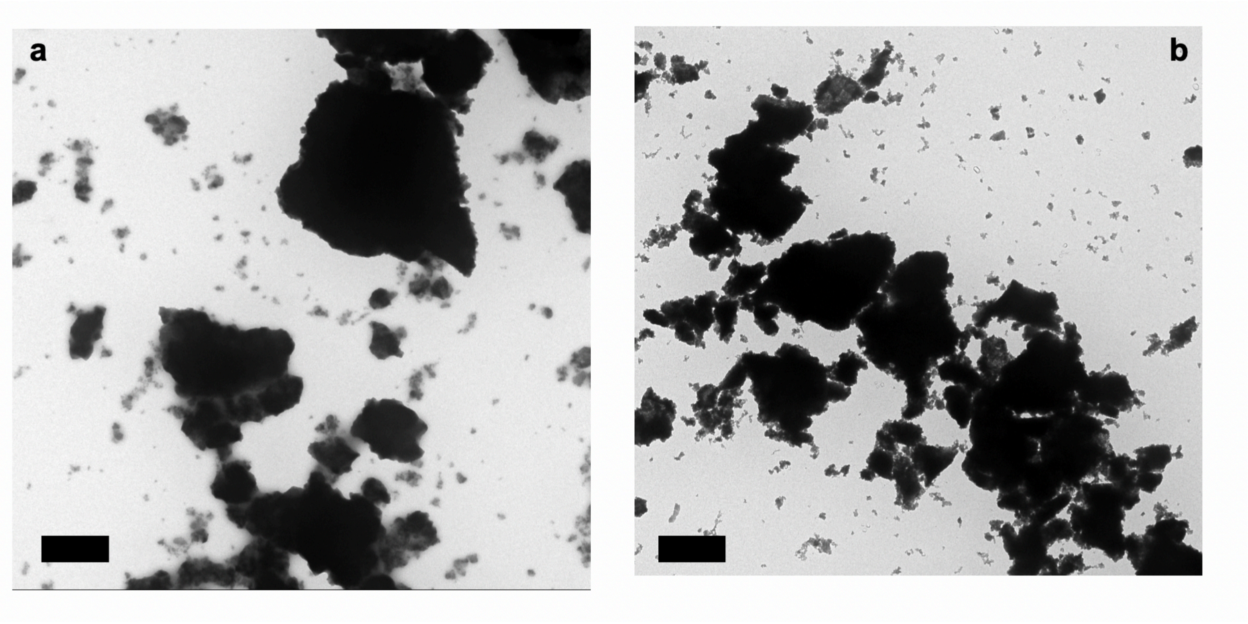

Supplementary Figure 15. Cycled metal-organic framework morphology. TEM images of (a) abiotic MIL-100 and (b) biotic MIL-100 after 10 Cr(VI) challenges. Scale bars are 1 μm.

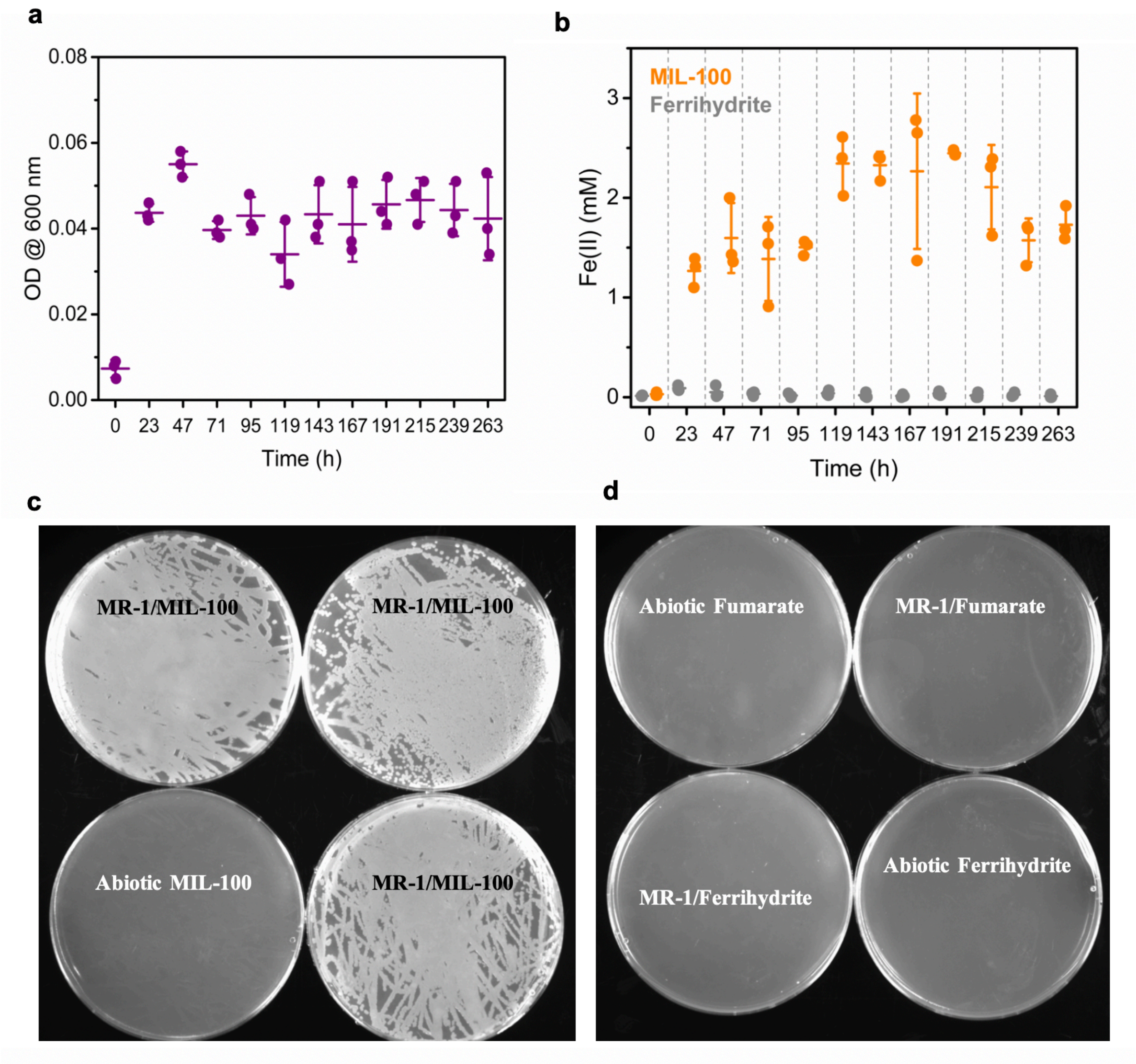

Supplementary Figure 16. Bacterial viability post-cycling. (a) Growth of MR-1 when grown on soluble fumarate (40 mM) over the course of the 10 Cr(VI) cycles. Data show mean ± S.D. for three independent biological replicates. (b) Fe(III) reduction of ferrihydrite (*grey*) and MIL-100 (*orange*) over the course of 10 Cr(VI) challenges. Data show mean ± S.D. for three independent biological replicates. (c-d) Bacterial viability assessed by plating on LB agar plates 23 h after the tenth 0.5 mM Cr(VI) challenge in MR-1/MIL-100, MR-1/Ferrihydrite, MR-1/Fumarate, and the abiotic samples. MIL-100 biotic samples are shown in triplicate, while only a single plate is shown for the others. Only MR-1/ MIL-100 samples exhibited growth. Source data for (a) and (b) are provided as a Source Data file.

Supplementary Tables

Supplementary Table 1. Cr(VI) cycles.

Cycle Number	Culture Volume (μL)	Volume of 500 mM Cr(VI) Added (μL)	Cr(VI) Removed (mg g^{-1}) Biotic MIL-100 mean ± S.D. n=3	Cr(VI) Removed (mg g^{-1}) Biotic Ferrihydrite mean ± S.D. n=3
**1**	1960	1.96	7.61 ± 0.09	3.28 ± 1.07
**2**	1842	1.84	15.49 ± 0.09	5.96 ± 1.25
**3**	1724	1.72	23.52 ± 0.09	4.59 ± 2.20
**4**	1606	1.61	31.40 ± 0.09	5.00 ± 1.48
**5**	1487	1.49	39.23 ± 0.09	4.25 ± 1.34
**6**	1369	1.37	47.26 ± 0.00	1.37 ± 2.08
**7**	1250	1.25	55.09 ± 0.09	5.55 ± 0.62
**8**	1131	1.13	62.97 ± 0.09	2.74 ± 2.25
**9**	1012	1.01	70.85 ± 0.09	5.21 ± 3.29
**10**	893	0.89	78.67 ± 0.09	5.14 ± 2.56

Supplementary Table 2. Cr(VI) removal by both biotic and abiotic agents.

Material	Cr(VI) Capacity (mg g^{-1})	Process	Reference
**Humic Acid Coated Magnetite Nanoparticles**	3.37	Fe Oxidation	1
**Bio amended Fe^0**	6.2	Fe Oxidation	2
**Ferrihydrite**	12.97	Fe Oxidation	3
**FIR-53**	17.8	Anion Exchange	4
**UV Modified Corn Straw**	20.04	-OH, $-CO_3$, -COOH Complexation	5
***Geobacter sulfurreducens*-Reduced Chlorite (+AQDS)**	20.8	Fe Oxidation	6
**Sulfamate-Bacterial Cellulose**	22.73	$-NH_3$ and $-OH_2$ Reactions	7
**Living *Bacillus coagulans* Biomass**	23.8	Biosorption	8
**Wheat Straw Biochar**	24.6	Surface Reduction	9
**FIR-54**	24.7	Anion Exchange	4
**Aluminum Substituted Ferrihydrite**	39.79	Fe Oxidation	3
**Dead *B. coagulans* Biomass**	39.9	Biosorption	8
**$ABT\cdot 2ClO_4$**	65	Anion Exchange	10
**MONT-1**	55.2	Anion Exchange	10
**HCl Modified Willow Residue+Fe(III)**	113.64	Adsorption	11
**Wheat Straw +FeS**	150	Adsorption/Fe Oxidation	12
**HCl Modified Willow Residue**	217.39	Adsorption	11
**Zn-MOF-74**	750	Fe(II) Surface Enhancement	13
**UTSA-74**	796	Fe(II) Surface Enhancement	13

Supplementary Table 3. Metal-organic framework surface areas.

Material	Measured Langmuir Surface Area (m^2g^{-1})	Reported BET Surface Area (m^2g^{-1})	Reference
**MIL-100**	2188 ± 25	1974	[14]
**Fe-BTC**	1512 ± 92	1092	[15]
**MIL-88A**	130 ± 3	25	[16]

Supplementary References

1. Jiang, W. *et al.* Cr(VI) Adsorption and Reduction by Humic Acid Coated on Magnetite. *Environ. Sci. Technol.* **48,** 8078–8085 (2014).
2. Yin, W. *et al.* Enhanced Cr(VI) removal from groundwater by Fe0-H2O system with bio-amended iron corrosion. *J. Hazard. Mater.* **332,** 42–50 (2017).
3. Ni, C. *et al.* Adsorption performance of Cr(vi) onto Al-free and Al-substituted ferrihydrites. *RSC Adv.* **6,** 66412–66419 (2016).
4. Fu, H.-R., Xu, Z.-X. & Zhang, J. Water-Stable Metal–Organic Frameworks for Fast and High Dichromate Trapping via Single-Crystal-to-Single-Crystal Ion Exchange. *Chem. Mater.* **27,** 205–210 (2014).
5. Peng, Z. *et al.* UV modification of biochar for enhanced hexavalent chromium removal from aqueous solution. *Environ. Sci. Pollut. Res.* **25,** 10808-10819 (2018).
6. Brookshaw, D. R., Coker, V. S., Lloyd, J. R., Vaughan, D. J. & Pattrick, R. A. D. Redox Interactions Between Cr(VI) and Fe(II) in Bioreduced Biotite and Chlorite. *Environ. Sci. Technol.* **48,** 11337–11342 (2014).
7. Lu, M., Guan, X.-H., Xu, X.-H. & Wei, D.-Z. Characteristic and mechanism of Cr(VI) adsorption by ammonium sulfamate-bacterial cellulose in aqueous solutions. *Chinese Chem. Lett.* **24,** 253–256 (2013).
8. Srinath, T., Verma, T., Ramteke, P. W. & Garg, S. K. Chromium (VI) biosorption and bioaccumulation by chromate resistant bacteria. *Chemosphere* **48,** 427–435 (2002).
9. Tytłak, A., Oleszczuk, P. & Dobrowolski, R. Sorption and desorption of Cr(VI) ions from water by biochars in different environmental conditions. *Environ. Sci. Pollut. Res.* **22,** 5985–5994 (2014).
10. Li, X., Xu, H., Kong, F. & Wang, R. A Cationic Metal-Organic Framework Consisting of Nanoscale Cages: Capture, Separation, and Luminescent Probing of Cr 2O 72−through a Single-Crystal to Single-Crystal Process. *Angew. Chem. Int. Ed.* **52,** 13769–13773 (2013).
11. Zhu, Y. *et al.* Removal of hexavalent chromium from aqueous solution by different surface-modified biochars_ Acid washing, nanoscale zero-valent iron and ferric iron loading. *Bioresource Technol.* **261,** 142–150 (2018).
12. Lyu, H. *et al.* Removal of hexavalent chromium from aqueous solutions by a novel biochar supported nanoscale iron sulfide composite. *Chem. Eng. J.* **322,** 516–524 (2017).
13. Luo, M. B. *et al.* The MOF +Technique: A Significant Synergic Effect Enables High Performance Chromate Removal. *Angew. Chem. Int. Ed.* **56,** 16376–16379 (2017).
14. Guesh, K. *et al.* Sustainable Preparation of MIL-100(Fe) and Its Photocatalytic Behavior in the Degradation of Methyl Orange in Water. *Cryst. Growth Des.* **17,** 1806–1813 (2017).
15. Sanchez-Sanchez, M., de Asua, I., Ruano, D. & Diaz, K. Direct Synthesis, Structural Features, and Enhanced Catalytic Activity of the Basolite F300-like Semiamorphous Fe-BTC Framework. *Cryst. Growth Des.* **15,** 4498–4506 (2015).
16. Wang, J. *et al.* Metal–organic frameworks MIL-88A with suitable synthesis conditions and optimal dosage for effective catalytic degradation of Orange G through persulfate activation. *RSC Adv.* **6,** 112502–112511 (2016).
